# Helping Peer Specialists Succeed: Lessons from a Statewide Training Program

**DOI:** 10.21203/rs.3.rs-9569288/v1

**Published:** 2026-05-09

**Authors:** Jeremy Fine, Mark Holmes, Helen Newton, Nathaniel Sowa, Amy Watson, Kristen Hassmiller Lich

**Affiliations:** University of North Carolina at Chapel Hill; University of North Carolina at Chapel Hill; University of North Carolina at Chapel Hill School of Medicine; University of North Carolina at Chapel Hill School of Medicine; Wayne State University; University of North Carolina at Chapel Hill

**Keywords:** peer support specialists, certified recovery support specialists, workforce development, training completion, certification, certification examination, program completion, student supports, academic accommodations, financial support, attrition, behavioral health workforce, mental health workforce, recovery support, Illinois

## Abstract

**Objective:**

The purpose of this study was to determine factors related to successful completion of peer support specialist training and certification.

**Methods:**

This study analyzed enrollment data to study graduation outcomes in 1,164 participants in the Certified Recovery Support Specialist (CRSS) Success Program, an intervention designed to scale up the number of peer specialists in the state by funding tuition and direct supports for students. Exploratory institutional-level binomial regression and grant funding data was used to understand the relationship between funding and graduation rate. Post-graduation survey data from 171 participants was also analyzed with logistic regression to understand factors associated with attempting and passing the peer specialist certification exam.

**Results:**

Receiving financial support beyond tuition and receiving academic accommodations were strongly associated with an increased likelihood of graduation, while requiring service supports like counseling or legal assistance decreased this likelihood. Working as a peer specialist during training also significantly boosted the probability of graduation, whereas taking a leave of absence did the opposite. Among graduates, receiving tangible support during their training and having a smaller gap between graduation and attempting the exam were associated with attempting and passing the exam, respectively.

**Conclusions:**

Direct supports for students are associated with successful progression through the peer specialist training pipeline. Workforce development programs like the CRSS Success Program can further optimize outcomes by providing additional supports, especially to students at-risk of attrition.

## Plain language summary

Peer specialists help provide recovery support to others living with mental health conditions or substance use disorders. Training more peer specialists is important because there is a shortage of mental healthcare providers in the US. This study analyzed data from a program that offered students scholarships and direct supports like financial help and accommodations to students. These supports were found to be very effective in helping students complete their training and certification.

## Introduction

The United States (US) mental health workforce does not have enough capacity to meet the needs of all its people,^1^ with over 122 million Americans living in behavioral health shortage areas.^2^ One partial solution is expanding the peer specialist workforce.^3^ Peer specialists, also known as peer support specialists, are individuals with lived experience of recovery from behavioral health conditions who use their experience to support others by providing strengths-based, empathetic support.^4–6^ They may help ameliorate the ongoing mental health workforce shortage by shifting non-clinical recovery support away from other providers.^7–10^ Recent evidence highlights the promising role peer specialists can play in helping clients feel empowered and maintain their recovery, and suggests they may even help reduce mental health service utilization among their clients.^11–18^ Given this evidence, the Substance Abuse and Mental Health Services Administration (SAMHSA) has recommended including peer specialists in several contexts, including across the mental health crisis continuum.^19^

Infrastructure for creating a peer specialist workforce has rapidly expanded since 2001.^20^ By 2024, nearly every state in the US offered a peer specialist certification,^21^ although there is significant variation by state in terms of classroom hours and on-the-job experience, many of which require fewer than 50 hours of didactics and over half of which do not require any experience prior to certification.^22,23^ Despite this expansion, there has been limited study of policies designed to grow the peer specialist workforce.^29^ This gap is particularly important given that training and certification have been associated with job satisfaction and retention among peer specialists.^24–26^ In states like Illinois (IL), certification may also help boost retention by increasing compensation, as certified peer specialists can bill Medicaid at a higher rate.^35^

The Certified Recovery Support Specialist Success Program (CRSS-SP) is a state-funded grant program in IL that allocates funds for institutions to operate peer specialist training programs at no cost for students. This program directs recipients to use their funding to help students overcome any barrier inhibiting their success, ranging from cash payments to help students afford basic needs to legal or counseling services. Students must complete at least 100 hours of course work and 300 hours of on-the-job internship experience to graduate. The program provides a stipend for those completing unpaid internships.^27^ Following graduation, students are eligible to sit for the certification exam offered by the non-profit certification board in IL.^28^ The CRSS-SP was designed to provide an alternative to the previous “independent pathway” for certification, through which individuals navigated the training process themselves, finding and enrolling in 100 hours of eligible course work and completing one year of peer specialist work in order to be eligible for certification.^28^ Even though the “independent pathway” had been available for 14 years, there were only 224 actively certified peer specialists in IL prior to implementation of the CRSS-SP.^27^

To understand factors associated with successful training and certification of peer specialists, this study analyzed administrative and outcomes data from the CRSS-SP.

## Methods

### Ethical Approval

This study was deemed exempt by the Institutional Review Board of the institution of the first author.

### Data Sources

#### Source 1: Enrolled-Student Data

Current student data is collected each term via surveys filled out by program administrators at each CRSS-SP site. The data window used in this study began in Fall 2021 (the first cohort of students) through the middle of 2025 (N=1,172 students ever enrolled). Across 12 sites, initial demographic and interest-related covariates were collected via surveys administered to students, and progress was tracked longitudinally and reported each term. Grant amount and school-level characteristics were merged in from public grant reporting,^30^ and the Integrated Postsecondary Education Data System (IPEDS) from the National Center for Education Statistics.^31^ Total grant allocation was divided by total enrolled student-terms within each institution to determine the average amount allocated per student per term. Regression equations are illustrated in **Table 1.**

### Source 1 Cleaning and Variable Transformation

Analyses excluded participants outside the analytic scope and records that were structurally invalid, empty, or clearly erroneous. One institution was excluded from Analysis 1 and 2 due to unreliable reporting, one additional institution was excluded from Analysis 2 due to recently beginning to enroll students, resulting in the inclusion of 11 and 10 institutions in Analyses 1 and 2. Four groups of “support” variables were created based on binary, per-term, variables (i.e. “Did the student receive X support this term”); these were operationalized as percentage of enrolled-terms a student received at least one of each category of support, to control for educational duration. Free-text analysis was used to categorize “other” responses into existing and novel binary variables. Groups of support variables are illustrated in the **Supplement**, alongside additional variable cleaning information. Program fees and tuition were excluded as a support, as these are inherent to the program.

Logical constraints were applied to coursework and internship hours, ambiguous internship reporting was resolved using predefined rules, and a small number of missing terminal outcomes were imputed using prespecified procedures (see **Supplement**). All variables were then standardized into analysis-ready longitudinal measures. Analyses were run without imputed participants when appropriate; excluding imputed participants did not meaningfully change the sign or magnitude of results.

#### Source 2: Post-graduation Survey

Graduates who graduated at least 6 months prior were sent an anonymous online survey that they could fill out for a $30 gift card. Of the 370 graduates who received this survey, 171 completed it (response rate = 46.22%). The same demographic variables were collected, as were training-related variables (institution attended, which supports an individual received while in training, whether they received academic credit, graduation timing, Likert-scale questions like assessing perceived program support, exam-related variables (whether they attempted and passed the CRSS certification exam, exam timing), and employment related variables (if they have worked as a CRSS, wage, number of hours worked per week). Outcomes of interest included attempting the exam and passing the exam.

### Source 2 Cleaning and Variable Transformation

Free-text responses describing current work settings were reviewed, sorted, and consolidated into analytic categories. Likert-style items were recoded into binary indicators reflecting any agreement versus no agreement. A summative program support variable was constructed based on endorsement of 11 distinct support types received during training, as per-term supports were not available. Responses provided in an open-ended “Other” field were reviewed and incorporated into this total. Program fees and exam related fees were excluded from the total supports, as these are inherent to the CRSS-SP. Supports were grouped into similar categories to those in the enrolled-student data analysis. Some categorical variables were collapsed because of small cell sizes. Reported graduation and exam dates were used to derive time-since-graduation and time-between-graduation-and-exam variables.

### Analytical Technique

All samples exhibited low (<3%) covariate missingness; therefore, complete-case analysis was used.^32^ Logistic regression was used for all student-level analyses. For Analysis 2, in which graduation rate ($\frac{n_{graduates}}{n_{graduates}+n_{discontinued}}$) was the outcome, binomial regression was used with a quasibinomial specification to account for overdispersion.^33^ Prior to modeling, multicollinearity was assessed using variance inflation factors and pairwise correlations; variables with correlations exceeding r=.5 were not included together, and all variance inflation factors were below 4. Robust standard errors were used in all models. Final models included prespecified covariates based on theory and prior literature, with additional variables retained when they improved model fit. Additional details, including a detailed missingness analysis, and details on model specification and robustness testing are present in the **Supplement**.

## Results

Characteristics from the enrollment, post-graduate survey, and institution-level datasets are presented in **Tables 2 and 3**. The enrollment dataset included 1,164 students across 11 institutions. Across observed terms, students received at least one support beyond tuition in approximately 63% of terms. Training characteristics differed significantly across training status groups, whereas demographic characteristics were largely similar. An institution-level average of $17,486 was allocated per student-term. In the post-graduate survey (n = 171), 79/171 respondents (46.20%) reported attempting the certification exam at an average of 6.6 months after graduation. Among exam attempters (n = 79), 61/79 (77.22%) reported passing.

### Analysis 1

**Figure 1:**
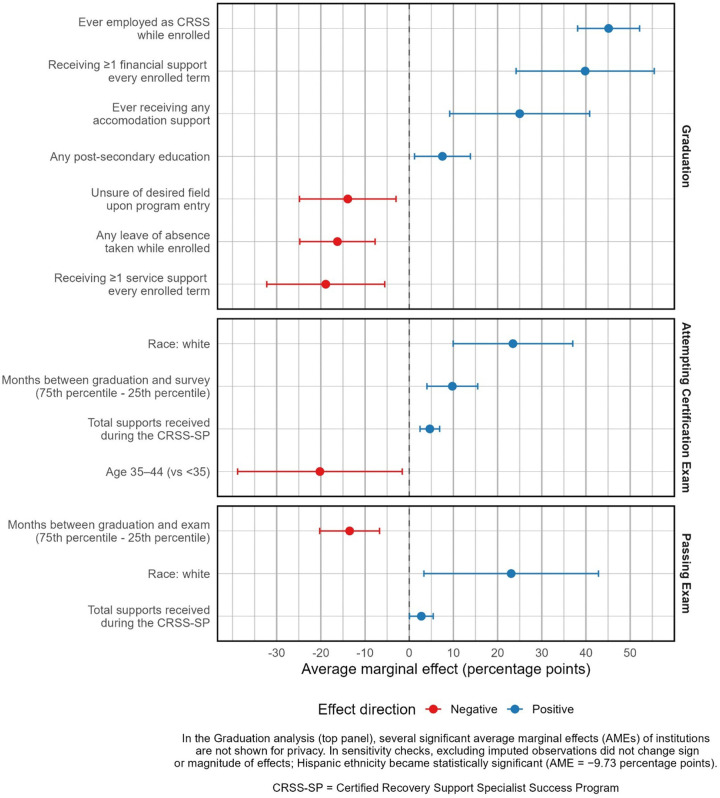
Average Marginal Effects of Significant Variables Across Analyses In the Graduation analysis (top panel), several significant average marginal effects (AMEs) of institutions are not shown for privacy. In sensitivity checks, excluding imputed observations did not change sign or magnitude of effects; Hispanic ethnicity became statistically significant (AME = −9.73 percentage points). CRSS-SP = Certified Recovery Support Specialist Success Program

The model showed strong discrimination (overall AUC = 0.86), with stable performance across 100 repeated 80/20 train–test splits (mean AUC = 0.83; 2.5th–97.5th percentile: 0.79–0.87). Probability of graduation was significantly higher among participants receiving financial supports, modeled as the proportion of enrolled terms in which at least one financial support was received (see the top graph of Figure 1). Receiving at least one financial support in every enrolled term corresponded to a +39.81 percentage point (pp) increase in the probability of graduation (95% CI: +24.17 to +55.44, p < .001); receiving financial support in half of enrolled terms would correspond to approximately +19.91 pp. Ever receiving accommodation support during training was also associated with a higher probability of graduation (+25.00 pp; 95% CI: +9.16 to +40.83, p = .002).

In contrast, receiving at least one service support in every enrolled term was associated with a lower probability of graduation (−18.89 pp; 95% CI: −32.24 to −5.54, p = .006). Employment as a peer specialist during training was associated with a substantially higher probability of graduation (+45.12 pp; 95% CI: +38.10 to +52.14, p < .001). Post-secondary education was associated with a modest increase (+7.51 pp; 95% CI: +1.18 to +13.83, p = .020), whereas being unsure of the intended field of practice (mental health, substance use disorder, or both) was associated with a lower probability of graduation (−13.90 pp; 95% CI: −24.83 to −2.98, p = .013).

### Analysis 2

**Figure 2:**
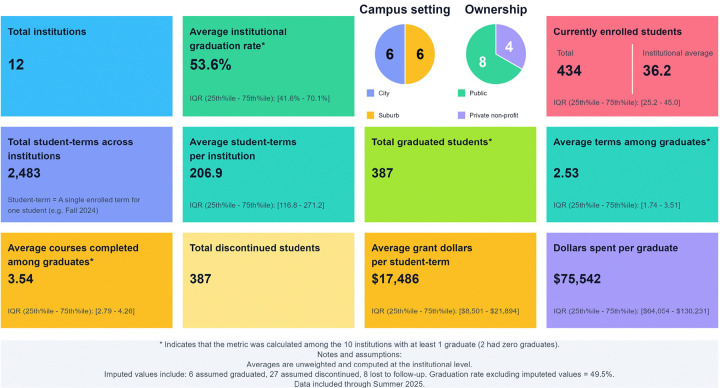
Descriptive Statistics on the Certified Recovery Support Specialist Success Program *Indicates that the metric was calculated among the 10 institutions with at least 1 graduate (2 had zero graduates). Notes and assumptions: Averages are unweighted and computed at the institutional level. Imputed values include: 6 assumed graduated, 27 assumed discontinued, 8 lost to follow-up. Graduation rate excluding imputed values = 49.5%. Data included through Summer 2025.

Across all 12 institutions, $29,234,739 has been invested in the CRSS-SP and the program has produced 387 graduates (Figure 2). The average institutional cost for each student-term is $17,486, and $75,542 to produce each graduate. 627 student-terms, or 26.21% of all student-terms, were attributed to 387 students who ultimately discontinued the program, representing $7,663,119 in expenditures. The average institutional graduation rate was 56.30% among the 10 schools with graduates.

Each additional $1,000 in grant spending per student-term was associated with a +2.03 percentage-point increase in institutional graduation rate (95% CI: +0.76 to +3.31 pp; p = .002). The estimated effect was directionally consistent and similar in magnitude across leave-one-out analyses, suggesting that, despite the small sample, the result was not driven by any single institution.

### Analyses 3 and 4

The model showed moderate discrimination (AUC = 0.77). In 100 repeated, 70/30 train–test splits, mean AUC was 0.73, with variability across spreads (2.5th–97.5th percentile: 0.60 to 0.88). Among the 171 graduates who responded to a follow-up survey, 103 are actively working as peer specialists, 79 have taken the state’s certification exam, and 61 have passed the exam. Each additional unique support received during training (of 13 total supports, including “Other”) was associated with a +4.67 pp increase in the adjusted probability of attempting the exam (95% CI: +2.44 to +6.89; p < .001), and each additional month since graduation was associated with a +2.91 pp increase (95% CI: +1.31 to +4.50; p < .001). The middle graph in Figure 1 depicts the effect of moving from the 25th percentile of time since graduation to the 75th percentile (8.73 months since graduation vs 11.95 months). Individuals aged 35–44 had a −20.22 pp lower adjusted probability of attempting the exam than those under 35 (95% CI: −38.85 to −1.58; p = .033), while identifying as White was associated with a +23.47 pp increase compared with non-White individuals (95% CI: +9.93 to +36.99; p = .001). Support-specific sensitivity analyses suggested the composite support effect was primarily attributable to tangible supports like technology and professional clothing (AME = +15.43 pp, 95% CI: +5.74 to +25.12; p = .002), with no evidence for financial or service supports in the grouped model. Replacing the summed support count with a technology support indicator improved model fit (AIC = 202.58 vs 207.23); predicted probabilities from the two models were highly correlated (r = 0.83), suggesting this support drives the effect in the model. To assess whether the association of White race with exam attempt was partly attributable to training program attended, the model was rerun on the subset with sufficient institutional data (14 observations excluded), with and without institution indicators. In this reduced sample, the estimated effect of White race decreased from +26.88 pp (95% CI: +12.36 to +41.28, p<.001) to +19.30 pp (95% CI: +0.24 to +36.17, p=.025) after institution indicators were added, suggesting that some portion of the association reflected differences in training program attended.

In the model investigating factors associated with passing the exam, the small sample size required outcome-stratified robustness testing with minimum numbers of each outcome in the testing and training sets (≥3 per class in the test set and ≥8 per class in the training set). Using 100 repeated 80/20 train–test yielded a mean AUC of 0.78 with a widely variable spread (median 0.79; 2.5th–97.5th percentile: 0.44 to 1.00). Each additional support was associated with a +2.74 pp increase in probability of passing (95% CI: +0.05 to +5.42, p = .046), being white was associated with a +23.07 pp increase (95% CI: +3.32 to +42.81, p = .022). Each additional month waited between graduation and taking the exam was associated with a −2.24 pp decrease likelihood of passing (95% CI: −3.24 to −1.24, p < .001).The bottom graph in Figure 1 depicts the negative impact of waiting approximately 6 additional months to take the exam, from the 25th percentile of exam timing to the 75th percentile (3.02 months vs 9.00 months).

## Discussion

The results suggest that the CRSS-SP is effectively adding trained peer specialists to the IL mental health workforce and may inform best practices for state peer specialist training pipelines. To boost training program completion, this study found support for offering students direct financial assistance, academic accommodations, and opportunities to work in peer specialist roles during training. Conversely, students who received support services like legal assistance or mental health counseling, those who took a leave of absence, did not receive post-secondary education, or unsure of what field they wanted to enter faced elevated risk of program discontinuation. This aligns with anecdotal reports that some early participants entered the CRSS-SP without a strong commitment to completion and later discontinued. In response, institutions reportedly revised admission standards.

While some degree of attrition is unavoidable, especially given that participants may face substantial challenges during their educational journeys,^34^ these findings suggest institutions may be able to reduce discontinuation by allocating existing resources more strategically. Exploratory evidence from this study also suggests that increasing per-term funding may boost graduation rates, although this requires confirmation with larger datasets.

This study also found factors influencing certification exam attempt and pass rates, such as offering technological support like laptops, tablets, and hotspots, during training, and encouraging exam attempts soon after graduation. White respondents were more likely to attempt and pass the exam, although is not clear whether this finding is explained by structural racism, or may represent confounding. When institution indicators were included, the effect of race decreased by almost 30%, suggesting that at least some portion of the racial effect was due to differences in training program attended.

The size of the certified peer specialist workforce in IL has grown by over one-fourth since students began graduating from the program in 2022. By decreasing barriers to certification, IL is creating a pool of trained peer specialists that may help address workforce shortages. The ability of certified peer specialists to bill Medicaid at a higher rate may help reduce the documented wage insufficiency among peer specialists, which is a known driver of dissatisfaction in the peer specialist literature.^36,37^ While the CRSS-SP is generating more certified peer specialists, there is still substantial leakage along the pipeline between graduation and certification. Among survey participants who worked as a peer specialist since completing the program, 52 of 115 (45.2%) had either not attempted the certification exam or had attempted and failed it. Closing this gap may be an area of interest for policymakers in IL.

Contextualizing the cost per CRSS-SP graduate in isolation is challenging, however, because the program covers start-up costs, tuition, and student supports, and there is no obvious strong comparator against which to benchmark that investment. Future studies may seek to use parameters generated from this study to perform cost-effectiveness analyses, which may be particularly valuable given that becoming a peer specialist is associated with increased employment and decreased Medicaid mental healthcare expenditures, potentially generating additional tax-revenue and decreasing healthcare costs paid by the state.^38–42^.

This research has limitations. The enrolled-student and post-graduation data sets used in this study were unlinked, so outcomes could not be tracked longitudinally at the individual level. Additionally, the lack of standardization in enrolled student data reporting required significant cleaning and outcome-status imputation. Although sensitivity tests showed similar findings without imputed observations, data entry errors from administrators may remain. Furthermore, there may be nonresponse bias in the post-graduate survey. The post-graduate data were not sufficiently powered to study significant factors associated with ever working as a peer specialist following graduation, and results derived from this sample have wide confidence intervals and should be interpreted with caution. Given that this study used observational data, significant findings such as the association of financial supports with graduation should not be interpreted as causal and may be impacted by confounding.

In terms of generalizability, other states choosing to adopt IL’s approach to peer specialist training may differ in the ability to implement the CRSS-SP model, given each state’s unique political, funding, and educational landscape. Even so, this study reveals numerous risk and protective factors for progression along the peer specialist training pipeline. Future program administrators should collect linked longitudinal data so future investigators can replicate these findings and determine factors associated with becoming a peer specialist after graduation. This should include precise measurement of the dose and exact nature of supports provided to each student to better isolate their impact on key outcomes. Finally, given the qualitative work exploring the barriers faced by peer specialists in the workplace,^24,43,44^ and documented long term retention concerns,^45,46^ future studies should evaluate whether interventions like the CRSS-SP affect peer specialist retention.

## Supplementary Material

Tables

Tables are available in the Supplementary Files section.

Supplementary Files

This is a list of supplementary files associated with this preprint. Click to download.

• Supplement1.docx

• Tables.docx
